# Development of a knowledge broker group to support evidence-informed policy: lessons learned from Myanmar

**DOI:** 10.1186/s12961-021-00806-x

**Published:** 2021-12-28

**Authors:** Pyone Yadanar Paing, Zarni Lynn Kyaw, Matthew Schojan, Tom Traill, Si Thura, Nilar Tin, Than-Tun Sein, Hnin Hnin Tha Myint, Paul Bolton, Catherine Lee

**Affiliations:** 1 Community Partners International, 81 University Avenue Road, Shwe Taung Gyar Ward (1), Bahan Township, Yangon, Myanmar; 2 Department of Mental Health, Global Mental Health Research Group, Johns Hopkins School of Public Health, 624 N. Broadway, Baltimore, MD United States of America; 3 Public Health Professional, Yangon, Myanmar; 4 Anthropology Department, Ramanya Hall, Yangon University, Yangon University PO, Yangon, Myanmar

**Keywords:** Knowledge translation platform, Evidence-informed decision-making, Health policy, Myanmar

## Abstract

**Background:**

Globally, policy-makers face challenges to using evidence in health decision-making, particularly lack of interaction between research and policy. Knowledge-brokering mechanisms can fill research–policy gaps and facilitate evidence-informed policy-making. In Myanmar, the need to promote evidence-informed policy is significant, and thus a mechanism was set up for this purpose. This paper discusses lessons learned from the development of the Knowledge Broker Group–Myanmar (KBG-M), supported by the Johns Hopkins Bloomberg School of Public Health’s Applied Mental Health Research Group (JHU) and Community Partners International (CPI).

**Methods:**

Sixteen stakeholders were interviewed to explore challenges in formulating evidence-informed policy. Two workshops were held: the first to further understand the needs of policy-makers and discuss knowledge-brokering approaches, and the second to co-create the KBG-M structure and process. The KBG-M was then envisioned as an independent body, with former officials of the Ministry of Health and Sports (MoHS) and representatives from the nongovernmental sector actively engaging in the health sector, with an official collaboration with the MoHS.

**Results:**

A development task force that served as an advisory committee was established. Then, steps were taken to establish the KBG-M and obtain official recognition from the MoHS. Finally, when the technical agreement with the MoHS was nearly complete, the process stopped because of the military coup on 1 February 2021, and is now on hold indefinitely.

**Conclusions:**

Learning from this process may be helpful for future or current knowledge-brokering efforts, particularly in fragile, conflict-affected settings. Experienced and committed advisory committee members enhanced stakeholder relationships. Responsive coordination mechanisms allowed for adjustments to a changing bureaucratic landscape. Coordination with similar initiatives avoided overlap and identified areas needing technical support. Recommendations to continue the work of the KBG-M itself or similar platforms include the following: increase resilience to contextual changes by ensuring diverse partnerships, maintain advisory committee members experienced and influential in the policy-making process, ensure strong organizational and funding support for effective functioning and sustainability, have budget and timeline flexibility to allow sufficient time and resources for establishment, organize ongoing needs assessments to identify areas needing technical support and to develop responsive corrective approaches, and conduct information sharing and collaboration between stakeholders to ensure alignment.

## Background

Globally, policy-makers face a difficult challenge in allocating resources to deal with problems affecting the population effectively [1]. With growing health demand, policy-makers need to prioritize allocation of scarce resources to meet population health needs, especially in low- and middle-income countries (LMICs). In deciding what issues to prioritize and how best to address these problems, one of the factors required is accurate and timely data on the nature and extent of issues and practical solutions. For instance, economic evaluation studies can provide rigorous data to inform and improve the healthcare decision-making process [15]. A second necessary factor is the skills and transparent processes to find out and use data effectively [1–4]. Although evidence-informed decision-making is quite new to LMICs, studies indicate a willingness on the part of policy-makers to embrace use of evidence in the health policy development process because it is seen to have the potential to play an increasingly important role in strengthening the health systems and deciding priority public health interventions [16]. However, health policy-relevant research is often designed and conducted with little input from local policy-makers and, therefore, may not sufficiently address policy-makers' informational needs [1, 5, 6, 13]. A review of the global literature on this issue suggests that this is a common problem: little interaction and understanding between policy-makers and researchers results both in policy-making that is not as well informed by data and research as it could be, and in research that does not address policy-makers' priorities [1, 4–6]. A systematic review of studies conducted mainly in LMICs of sub-Saharan Africa, Central America and the Middle East reported that the main barriers to the use of data by policy-makers in decision-making included a lack of research availability, lack of relevant research, having no time or opportunity to use research evidence, lack of skill among policymakers and other users in research methods, and costs [1]. The findings of a multi-country study conducted with policy-makers in Argentina, Egypt, Iran, Malwi, Oman and Singapore showed that one of the substantial barriers in evidence-informed decision-making was poor communication between researchers and policy-makers and lack of a proper channel to disseminate research results [17].

Based on interviews with policy-makers and researchers, one significant recommendation from the global literature concerns the need for more interaction between policy-makers and researchers [6]. This entails not just single meetings, but a process of regular or irregular meetings in which information is shared and where policy-makers have input into designing research relevant to them and researchers can share their knowledge and expertise with policy-makers in ways that the policy-makers find helpful [6, 7]. Knowledge-brokering mechanisms are a promising strategy to fill this research–policy gap and foster optimal use of research findings and evidence in policy-making [7]. Globally, knowledge translation platforms or knowledge-brokering mechanisms have been put in place to link health research with policy development and implementation. Examples of these come from Evidence-informed Policy Networks (EVIPNet) established by WHO to promote the systematic and transparent use of health research evidence in policy-making, focusing on LMICs. [8]. Evaluations have shown that many knowledge translation platforms/knowledge-brokering mechanisms have contributed to increased awareness of the importance of evidence-based decision-making and strengthened relationships among policy-makers, stakeholders and researchers [9].

With Myanmar’s commitment to achieving universal health coverage (UHC) by 2030, the need to promote evidence-informed policy has become increasingly important [10]. This need was recognized by Johns Hopkins University (JHU), Community Partners International (CPI) and the Ministry of Health and Sports, and was the basis for this project. JHU and CPI had taken initiatives to organize a knowledge broker group in Myanmar with the funding support of the United States Agency for International Development (USAID) through the Victims of Torture Fund (Award No. AID-OAA-LA-15–00003).

In addition to the CPI-JHU effort in Myanmar, knowledge translation initiatives have been attempted by international organizations in collaboration with the Myanmar Ministry of Health and Sports (MoHS). The Data to Policy (D2P) training course (2019–2020) implemented by the Bloomberg Philanthropies Data for Health Initiative aimed to build MoHS staff's capacity to use data and analytical methods to develop new policy recommendations [11]. The Department of Medical Research (DMR)’s collaboration with WHO’s Special Programme for Research and Training in Tropical Diseases (TDR) has conducted operational research capacity-building through Structured Operational Research and Training Initiative (SORT IT) courses since 2015, in which one module was developing policy briefs [12, 14]. DMR also conducted a Translating Research into Policy and Practice (TRIP) training workshop in 2019 to strengthen the capacity of programme staff, researchers and academics to translate research evidence into policy and practice.

## Methods

In 2015, JHU started to interview policy-makers in Myanmar with the aim of understanding their needs as related to evidence-informed decision-making. The process used by JHU was based on the EVIPNet materials and advice from individuals involved in existing EVIPNet platforms. The first step was exploration of needs through individual in-depth interviews with key public health policy-makers. A total of 16 interviews were conducted with advisors to the MoHS, country representatives of both international and national nongovernmental organizations (NGOs), and directors or secretaries of community-based organizations.

Then, JHU collaborated with CPI, which was working with the MoHS on activities related to UHC and Myanmar’s National Health Plan (NHP). Together with cooperation from CPI and the NHP Implementation Monitoring Unit (NIMU) of the MoHS, a series of workshops were organized in Nay Pyi Taw to support the development of a knowledge broker system and to understand the needs of in-country researchers to participate in this knowledge broker system. The structure of the workshops was a combination of presentations and working subgroup interactive discussions.

The first workshop was organized in November 2017, with the objectives to understand the needs of Myanmar policy-makers in formulating evidence-informed policy, describe approaches to formulating evidence-informed policy and familiarize the role of research in informing policy. It was attended by a total of 38 stakeholders from the MoHS, NGOs, universities, international donors and a private organization. The second workshop was organized in April 2018, with the aim of identifying the structure for a knowledge broker group that could support evidence-informed health policy in Myanmar and formulate a process and parameters for developing such a system. A total of 40 participants from the MoHS, NGOs, university and international donors attended the workshop.

The commitment from the stakeholders in the workshop led to taking steps to formally establish the Knowledge Broker Group–Myanmar (KBG-M), including the formation of a development task force which served as an advisory committee and a series of advisory committee meetings. Advisory committee members were recruited through the CPI and JHU existing networks and included senior public health advisors who were former officials of the MoHS and representatives from NGOs and civil society organizations (CSOs) actively engaging in the health sector, particularly in research and policy components. CPI and JHU focused on inviting individuals involved in developing the NHP (2017–2021), which was the first-ever strategic direction toward UHC in Myanmar. Their participation as an advisory committee member was voluntary, and commitment was maintained by their passion, desire and vision for facilitating evidence-informed policy in Myanmar. They were highly committed to the development process in terms of contributing their expertise and coordinating with the MoHS, as they were champions of the country’s health system and had been involved in public health decision-making for many years, with a strong willingness to improve it.

The structure of the KBG-M was designed to have a leader group—Board of Chairs (BoC)—and a Core Group (CG). While the CG would be the working group for knowledge translation, policy advocacy and communications, BoC members would provide overall guidance and advice to the CG. The primary role of the KBG-M would be to facilitate evidence-informed policy in Myanmar by developing policy briefs, organizing policy dialogues, linking researchers and policy-makers, and answering policy questions. The initial approach was to form the KBG-M with membership from both the MoHS and NGO professionals, with the assumption that involving government policy-makers in the group would enhance KBG-M’s role in facilitating policy advocacy. However, the MoHS Minister requested that all MoHS staff be removed from the proposed list of direct members of the KBG-M to reduce any conflict of interest. With this change, the Minister agreed to have the KBG-M formed as a technical affiliation to the MoHS. Following this, the advisory committee continued engagement with MoHS for signing the technical agreement. Given this agreement from the MoHS, the task force decided to organize KBG-M as an independent body while having an official collaboration with the MoHS. In terms of thematic areas, it was decided that KBG-M would cover a wide range of topics under a broad public health umbrella, especially the areas which would be identified as foundations for UHC, instead of focusing on only a few specific topics.

Most of the meetings took place in Yangon, with submission of administrative documents to Naypyitaw. However, starting in early 2020, communication shifted to primarily remote due to COVID-19 restrictions. Each advisory committee member took responsibility for communication with respective MoHS stakeholders, leveraging personal relationships to be more effective. Secretariat members from CPI took charge of making appointments and organizing meetings and follow-ups, as well as financial management, since CPI managed the funds from USAID to support KBG-M activities.

## Results

In the initial in-depth interviews, the needs of policy-makers expressed included (1) technical assistance from researchers, (2) leadership skills training, (3) more data on issues related to health, and (4) input from the community and partners. They also described challenges to creating policy, including (1) the current top-down policy-making process, so that policy does not derive from evidence or input from knowledgeable individuals in the field; (2) lack of a process for communication between policy-makers and researchers. preventing access to existing relevant data and planning of appropriate research to fill data gaps; and (3) varying opinions about what the policy process should look like in terms of who gives input, how decisions are made, and the level of engagement needed from policy-makers and key stakeholders.

In the first workshop, the participants showed a clear interest in learning and using methods for formulating evidence-informed policy. They also recognized the need for better communication mechanisms between researchers and policy-makers through a knowledge broker group and the need for synthesized evidence and knowledge to fill policy gaps. The request was for communication to be more frequent and for there to be better summaries of information that more closely fit with the focus/needs of policy-makers. During the second workshop, the stakeholders developed a proposed structure for the KBG-M and prioritized the possible roles of the KBG-M based on other countries’ experiences.

These workshops led to the formation of the Development Task Force in October 2018, a 10-member advisory committee whose terms of reference (TOR) focused on establishing the KBG-M. Members included senior public health advisors who were former officials of the MoHS and representatives from NGOs and CSOs actively engaging in the health sector. This advisory committee met approximately monthly to work on establishment of the KBG-M.

From 2018 until early 2021, advisory committee meetings were organized, and steps were taken to formally organize the KBG-M, including seeking official recognition from the MoHS. Along with the advisory committee's formation, CPI staff members of the advisory committee took on the role of secretariat to coordinate both within the advisory committee and between the advisory committee and external stakeholders.

From August 2019 to October 2019, the advisory committee engaged with the key MoHS officials, including the Minister, to explain the KBG-M concept and seek their opinion and commitment towards establishing the KBG-M. Feedback from those officials was positive, with the Minister, in particular, providing positive remarks and requesting that the advisory committee formally submit the KBG-M proposal to the MoHS.

Therefore, TOR were prepared and submitted to the MoHS in November 2019. The Minister expressed his belief in the importance of an intermediary organization such as the KBG-M to support the Myanmar health system. In accordance with his request to proceed with the official submission process, the advisory committee prepared and submitted a concept note detailing the KBG-M scope and activities to the International Relations Department (IRD) of the MoHS in May 2020. At the same time, the advisory committee also engaged with potential KBG-M members who were senior officials from the MoHS, university rectors and representatives from leading NGOs and professional bodies. The committee also engaged with stakeholders from similar initiatives, such as focal points for the D2P programme and TRIP trainings, to discuss how the KBG-M could collaborate with existing initiatives and fill gaps.

All potential members agreed to be involved in the KBG-M. The concept note was revised and submitted again to the IRD according to their comments. In September 2020, the Minister officially approved the concept note for the KBG-M. However, upon his request, all MoHS staff were removed from being directly involved as members of the KBG-M to reduce any conflict of interest. Instead, the Minister agreed to have the KBG-M formed as a technical affiliation to the MoHS by assigning a deputy director general from IRD to serve as a direct focal point from the MoHS to the KBG-M. Spaces left vacant upon the removal of MoHS officials were filled by members of the advisory committee who were nominated by the advisory committee and agreed to volunteer.

In October 2020, the advisory committee successfully submitted the revised documents for a final technical cooperation agreement (TCA) with the MoHS. Revisions included noting that representatives from local organizations working on peace and conflict resolution should be included in the KBG-M given their significant contribution to the health policy process in Myanmar. In addition, advisory committee members identified the involvement of international technical advisory experts as being important to the KBG-M establishment and long-term sustainability; therefore, this was added to the TCA document, and preparatory steps were also taken to engage technical assistance from the K2P Center at the American University of Beirut, Lebanon. This link-up with international experts was essential, because the K2P Center was a member of EVIPNet and thus could facilitate more international linkage as a way of building practice from that platform and provide technical assistance to the KBG-M by sharing their experiences and expertise on how KBG-M should be developed and should function, including exploring potential funding resources.

The TCA signing process was expected to be completed quickly (in October 2020), according to an informal discussion between an advisory committee member and the Minister. However, there were delays because of lengthy official procedures due to COVID-19 and IRD’s additional steps to request comments from all departments under the MoHS.

Comments were only received in the second week of December, most of which were positive, agreeing with the terms of the TCA with only minor suggestions. IRD revised the TCA accordingly and submitted it to the Minister for his final review. In early January 2021, the Minister approved the TCA, and IRD did a final check of legal obligation in the agreement. In the last week of January 2021, the Attorney General's office confirmed that there was no legal obligation in the TCA, and it was sent back to IRD. Although there was a plan to follow up with IRD in early February, the process stopped because of the military's unexpected takeover of state power on 1 February 2021. Following this, most civil servants, including MoHS staff, became involved in the Civil Disobedience Movement and left their positions in protest of the military coup. As the formal health system under the MoHS stopped functioning after 1 February 2021, the process is now on hold indefinitely.

## Discussion

Initial steps taken to form the KBG-M, including interviews, discussions, workshops, establishing the advisory committee and seeking technical agreement with the MoHS, encompassed a period of 5 years (late 2015 to early 2021). While this was longer than we had anticipated or understood it would be from similar initiatives, there were several necessary steps, along with unexpected events, that slowed the process. One example of a necessary step was the coordination of the various meetings and workshops, which took a great deal of time, but were necessary to ensure that appropriate and relevant individuals were included. These workshops formed the base of the KBG-M development and provided a unique opportunity for stakeholders not only to learn about other similar platforms, but to have a facilitated way in which to discuss, debate and ultimately decide on the utility of a knowledge translation platform in Myanmar and its structure. In addition, invitations to individuals to join the advisory committee took time and slowed the process, but were necessary to make sure appropriate and relevant individuals were included. Involving a diverse group of people whose support was needed was a challenge. In addition, there was a need to form a small, committed and diverse group of the advisory committee to help move this process forward.

Having advisory committee members with strong commitment and extensive expertise played a vital role throughout the process. Advisory committee members included former senior public health officials and representatives from NGOs and CSOs influential in the Myanmar health system. Their reputation and strong relationships with senior public health officials from MoHS contributed considerably to convincing key stakeholders of the need for the KBG-M and accelerating the necessary steps in seeking approval. In addition, having a committed and well-organized secretariat group from CPI was essential to facilitating effective communication with key stakeholders and ensuring the provision of necessary administrative support to the advisory committee.

The KBG-M was intended to be an autonomous or semi-autonomous body, but an official communication channel with the MoHS was seen as important, so commitment from high-level policy-makers such as the Minister was essential. This level of commitment would ensure that the Minister assigned a department focal person to collaborate with the advisory committee, providing that critical link. However, attempting to obtain this commitment from the MoHS brought challenges and delays. Being closely aligned with the MoHS was eventually prioritized by the local stakeholders during the workshops because of the legitimacy it would confer to the KBG-M, although advisory committee members continually considered the pros and cons of alternative, more independent forms of institutionalization.

One of the key lessons learned from the process is that a knowledge-brokering mechanism designed to collaborate with government officials will need a coordination mechanism that can effectively respond to changing political dynamics. Despite a smooth transition in communication from NIMU to IRD, the need for a concrete plan to respond to political changes is a clear issue for future steps. Most importantly, overly investing in and having success contingent upon one stakeholder, in this case the MoHS, was a barrier to the establishment and sustainability of the KBG-M, which might have been avoided with a more diverse and equitable partnership. Because political crises and other unforeseen events can impact initiatives such as the KBG-M, it is necessary to have contingency plans in place regarding how the formation and functioning of the KBG-M can continue even under such circumstances.

As stated by many MoHS officials and advisory committee members, the concept of the KBG-M had a potential conflict of interest with similar initiatives. Coordination with relevant stakeholders in other initiatives enabled the KBG-M to avoid duplications of effort with these similar mechanisms and increased KBG-M’s ability to fill key gaps. Once it is established, we believe that the KBG-M will be able to collaborate with them by offering a communication platform to deliver their knowledge translation products to policy-makers, for which more in-depth consultations with relevant groups would be needed to design such an efficient knowledge broker system.

Finally, JHU’s existing network of health policy experts and the development of a relationship with international institutions such as the K2P Center paved the way for technical assistance to the KBG-M. However, it is important to identify areas that could be strengthened by outside technical support early on, and to bring those advisors and technical experts into the process earlier rather than later. In addition, it is essential to seek out technical experts who can complement the existing expertise in the advisory committee and the KBG-M, and that recognize and wish to support a KBG-M that is wholly owned by local stakeholders.

Some key lessons learned were related to unanticipated events, including natural disaster and significant political changes, which resulted in recognition of the need to have response mechanisms in place to overcome challenges resulting from those types of situations. COVID-19-induced travel restrictions hindered effective communication and advocacy with high-level stakeholders from the MoHS, because regular communication with MoHS officials was conducted via phone or online meetings, which was less effective and more challenging than in-person communication. On the other hand, remote communication through online meetings essentially improved communication and access to the advisory committee members, as online meetings allowed people to join from their own location and increased their availability to be more involved on a regular basis. Finally, the unexpected February 2021 military coup made it impossible to start the KBG-M as planned and highlighted the importance of preparation to overcome challenges in those situations.

### Recommendations

Utilizing these lessons learned during the process of establishing the KBG-M to date, the following are key recommendations to assist continued work on the KBG-M itself or similar knowledge broker platforms in the future.

- To increase resilience to changes in political structures and priorities, ensure that the platform is established and maintained through diverse partnerships that take into account a variety of stakeholders, paying particular attention to CSOs and considering different options of institutionalization.
- As was essential to the work described in this document, maintain advisory committee members who have experience with and are influential in the policy-making process in order to facilitate effective advocacy.
- For increased effectiveness of functioning and longer-term sustainability, strong attention should be paid to organizational buy-in to support the platform, as well as donor funding and human resources to sustain the platform.
- Because the process for establishing knowledge translation platforms is known to take time, it is essential that there is flexibility regarding the budget and timeline to allow sufficient time and resources for the overall establishment of the platform.
- Since the development process may be impacted by unforeseeable events, preparations should be taken to have contingency plans in place to overcome challenges from those situations.
- Initial and ongoing needs assessments are necessary to identify areas where expert technical support can most effectively support the platform and its work with policy-makers, to avoid duplicate work and waste of resources, and to serve as benchmark measures for responsive corrective approaches that strengthen the development and functioning of knowledge translation platforms.
- Information sharing and collaboration between the platform, similar initiatives and other stakeholders should be conducted to ensure alignment and coordination.

## Conclusions

Evidence-informed policy is essential for Myanmar’s low-resource setting to achieve UHC. There were previous knowledge translation initiatives prior to the KBG-M to promote evidence-informed decision-making; however, most of them were related to short-term capacity-building and knowledge-sharing activities. The KBG-M concept was the first of its kind to facilitate evidence-informed policy by establishing an intermediary mechanism as an ongoing system for broader scope on health issues. That is why several challenges, especially delays and unanticipated steps in seeking official agreement with the MoHS, were encountered throughout the process, along with opportunities and achievements.

Despite the advisory committee and secretariat team’s close coordination, the unanticipated effects of the COVID-19 global pandemic certainly delayed ministerial procedures further. The 1 February 2021 military coup prevented the signing of an official agreement of the KBG-M with the MoHS. These represent two exceptional obstacles that could not be overcome within the current model of the KBG-M. Progress has nevertheless been made. Myanmar public health policy-makers have recognized the need for an intermediary mechanism between research and policy to promote evidence-informed decision-making. This role could have been fulfilled by the KBG-M once it was established and may be demanded again when political stability returns.

Despite the fact that the KBG-M establishment has not yet been fully accomplished due to current political instabilities, experiences from the development process provide insights to guide future KBG-M work itself or other similar initiatives, both in Myanmar and abroad.

Given the recent political instability, the KBG-M advisory committee decided to organize the KBG-M with representatives from NGOs, CSOs and ethnic health organizations (EHOs) who are actively engaged in public health programmes. Instead of the MoHS as the target audience, the KBG-M will aim to engage and work directly with NGOs, EHOs, CSOs and development partners who make programming and funding policy decisions. KBG-M will also work with other knowledge translation initiatives, including the newly formed Communities of Practice led by CPI, to gather updated policy interests and questions and, ultimately, provide policy recommendations.

## Data Availability

Not applicable.

## Abbreviations

CPI: Community Partners International
CSO: Civil society organization
D2P: Data to Policy initiative by Bloomberg Data for Health Initiative
EHO: Ethnic health organization
EVIPNet: Evidence-informed Policy Network
IRD: International Relations Department, Ministry of Health and Sports
JHU: Johns Hopkins University
K2P Center: Knowledge to Policy Center at the American University of Beirut, Lebanon
KBG-M: Knowledge Broker Group–Myanmar
MoHS: Ministry of Health and Sports
NGO: Nongovernmental organization
NHP: National Health Plan
NIMU: National Health Plan Implementation Monitoring Unit
SORT IT: Structured Operational Research and Training Initiative
TCA: Technical cooperation agreement
TRIP: Translating research into policy and practice
UHC: Universal health coverage
WHO TDR: WHO Special Programme for Research and Training in Tropical Diseases
